# The utility of integrating nanopore sequencing into routine HIV-1 drug resistance surveillance

**DOI:** 10.1099/mgen.0.001375

**Published:** 2025-03-20

**Authors:** Daniel Bugembe Lule, Deogratius Ssemwanga, Pontiano Kaleebu, Damien C. Tully

**Affiliations:** 1 Department of Infectious Disease Epidemiology, London School of Hygiene and Tropical Medicine, Keppel Street, London WC1E 7HT, UK; 2 Medical Research Council/Uganda Virus Research Institute & London School of Hygiene and Tropical Medicine Uganda Unit, Plot 51-59 Nakiwogo Road, P. O. Box 49, Entebbe, Uganda; 3 St. Georges University of London, Cranmer Terrace, Tooting, London SW17 0RE, UK; 4 Uganda Virus Research Institute, Plot 51-59 Nakiwogo Road, P. O. Box 49, Entebbe, Uganda; 5 Centre for Mathematical Modelling of Infectious Diseases, London School of Hygiene and Tropical Medicine, London, UK

**Keywords:** drug resistance, HIV, nanopore sequencing, next-generation sequencing

## Abstract

HIV continues to be a significant global public health concern. In 2022, an estimated 29.8 million people living with HIV received antiretroviral treatment (ART). From this, an estimated 10–15% of individuals living with HIV have drug-resistant strains of the virus. Testing for resistance to antiretroviral drugs is recommended before initiating ART. However, such services are often inaccessible due to costs and the need for complex laboratory infrastructure. The assessment of HIV drug resistance (HIVDR) relies on genotyping sequencing and algorithms to interpret genotypic resistance test results. Genotypic assays involve Sanger sequencing of the reverse transcriptase (*RT*), protease (*PR*) and integrase (*IN*) genes of circulating RNA in plasma to detect mutations that are known to confer drug resistance. While state-of-the-art sequencing technologies have swept the globe and enhanced our global pandemic response capabilities, they are still sparingly used for HIVDR surveillance. The scale-up of ART, especially in low- and middle-income countries, necessitates the establishment of cheap, expeditious and decentralized methods for HIVDR monitoring. Here, we outline how one low-capital next-generation sequencing platform, namely, nanopore sequencing, could augment efforts in expanding HIVDR surveillance efforts, especially in resource-limited settings. We discuss that because of its versatility, nanopore sequencing can accelerate HIVDR surveillance in conjunction with scaling up ART efforts and outline some of the challenges that need to be considered before its widespread and routine adaptation to detect drug resistance rapidly.

**Impact Statement**

Drug resistance remains a global challenge in combatting the HIV pandemic. Traditional sequencing methods for monitoring drug resistance often fall short in low-resource settings due to high costs and infrastructure demands. Recently developed third-generation sequencing technology offers a significant advance in testing for drug resistance as current guidelines recommend and for public health surveillance. This perspective explores the historical role of sequencing in interpreting genotypic resistance and outlines how nanopore sequencing could expand resistance surveillance, particularly in resource-limited settings. By enabling decentralized testing, improving the detection of low-frequency variants and fostering data sharing through standardized bioinformatics pipelines, this technology could not only address current surveillance gaps but also establish a foundation for equitable access to drug resistance testing.

## Data Summary

No data were produced arising from this perspective.

## Introduction

To date, no functional cure exists that eliminates HIV from all body compartments, despite the availability of various antiretroviral medications that suppress the virus and thus delay the onset of HIV-related illnesses, including opportunistic infections [1,2]. Antiretroviral treatment (ART) remains the most effective way of reducing mortality and morbidity as well as reducing transmission by lowering the plasma viral load (PVL) [3,4]. The emergence of HIV drug resistance (HIVDR) is of great concern as this gives rise to variants capable of replicating in the presence of drugs. Drug-resistant variants usually continue to acquire mutations, further diverging from the drug-susceptible wild-type, a process that further reduces ART efficacy. HIVDR can develop through several mechanisms, with most resistance emerging *de novo* due to the error-prone viral replication cycle of the HIV polymerase, which leads to every possible point mutation in the HIV-1 genome occurring [5]. In individuals undergoing ART, the drugs exert selective pressure on HIV, allowing resistant variants to thrive. The transmission of resistance or infection with a drug-resistant HIV strain is also possible. Monitoring of HIVDR identifies major resistance mutations that may reduce the susceptibility of circulating viruses to the drugs within the administered combinations. Regimens may differ across countries due to local public health policies, although World Health Organization (WHO) guidelines are usually adhered to, though not always. This causes a variation in HIVDR trends across geographical locations, particularly for HIV integrase (IN)-targeting drugs like Dolutegravir (DTG) [6], which were not widely used in the developing world until recently [7,9]. To meet the 2030 UNAIDS 95-95-95 targets [10], reliable, expeditious and cost-effective methods for HIVDR analysis are crucial.

**Fig. 1.**
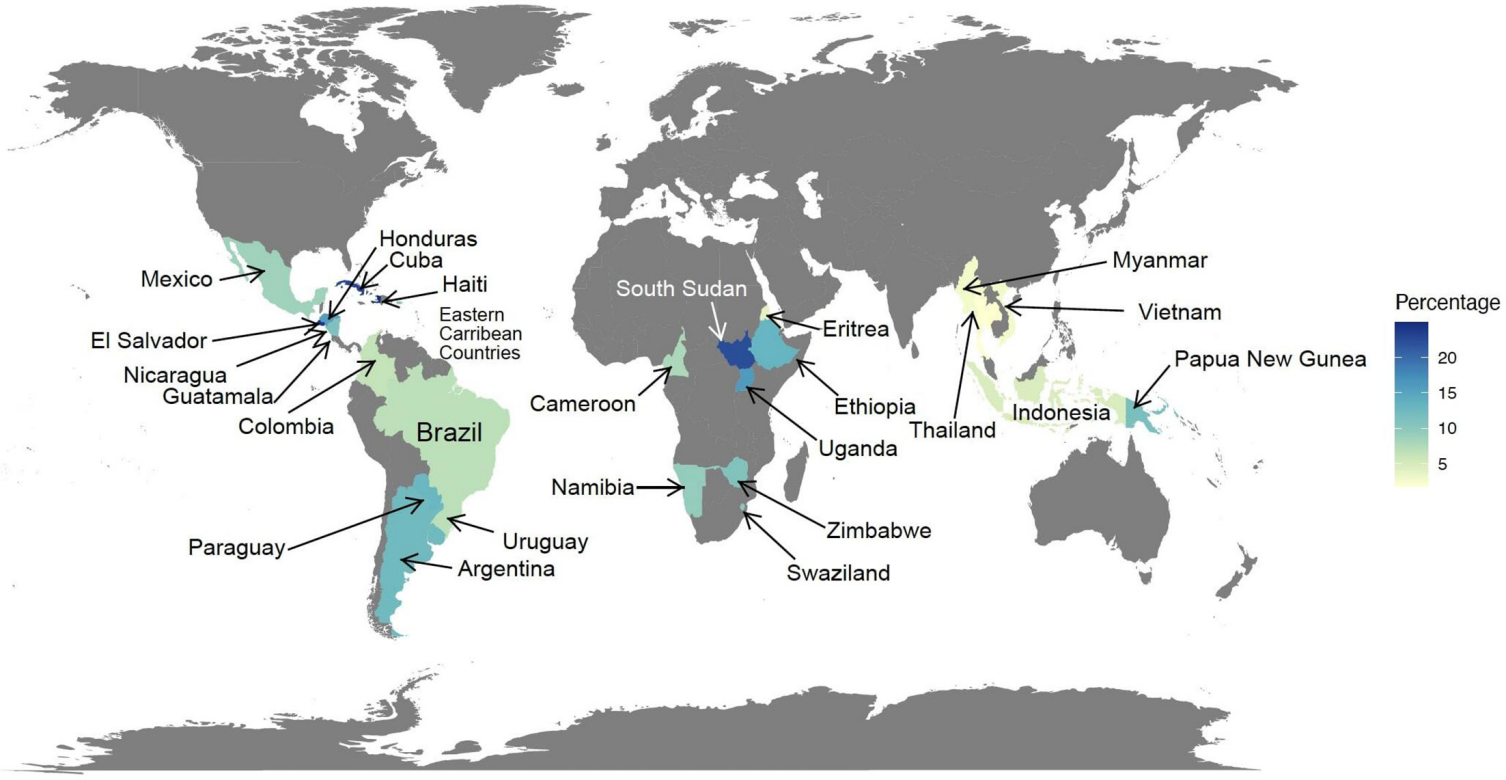
Prevalence of pre-treatment HIVDR. The map shows PDR reported for ART unexposed people by the WHO 2021 report [11], estimated to be ~10% based on median percentages for data from clinical trials of 21 national surveys.

HIVDR increased globally following the large-scale global dissemination of ART, and the WHO estimates it to be 10% for first-line drugs in their 2021 report, which was derived from the analysis of 21 national surveys [11] (Fig. 1). This is supported by various studies completed in African countries within the same period estimating pre-treatment HIVDR (PDR) at 11% [12]. A European meta-analysis that utilized the EuResist Integrated Database evaluated the trends of both transmitted and acquired HIVDR from 1981 to 2019 and reported a relatively similar rate for PDR of 13% and a high 68% for acquired HIVDR to any drug [13]. The high rate of acquired HIVDR in Europe could perhaps be a consequence of the long history of ART use and the ensuing longer survival periods of people living with HIV. Considering the extensive roll-out of ART globally, similar trends will likely be found in other geographical locations [14]. Treatment guidelines typically follow a structured approach, with most settings initiating therapy using first-line regimens composed of two nucleoside reverse transcriptase (RT) inhibitors (NRTIs) and one IN strand transfer inhibitor (INSTI). When treatment failure occurs, second-line regimens are introduced, which often consist of combination therapies, including INSTIs, NRTIs or non-nucleoside RT inhibitors (NNRTIs) and protease (PR) inhibitors (PIs) [15]. The decision to switch to second-line therapy is frequently guided by the HIVDR mutation profile. The WHO recommends switching to DTG-based regimens when resistance exceeds 10% [16,17]. However, recent data show DTG resistance emerging at higher-than-expected levels, ranging from 3.9 to 19.6% in low- and middle-income countries (LMICs), underscoring the need for both expanded HIVDR surveillance [18] and the development of a broader range of antiretroviral drugs and drug targets, extending beyond the traditional HIV-1 pol-targeting regimens [19]. A notable advancement in the field is the recent approval of Lenacapavir, a first-in-class HIV capsid inhibitor, by the European Union and the USA in August and December 2022, respectively [20,22]. This approval has significant implications for HIVDR surveillance, which has traditionally focused on the *pol* gene. Lenacapavir has demonstrated promising efficacy in clinical trials, achieving viral suppression to below 50 copies ml^–1^ in nearly 90% of participants within 15 days in a phase Ib study [23]. A subsequent 52-week study showed a sustained reduction in viremia to less than 50 copies ml^–1^ in 80% of participants [24]. As a next-generation antiretroviral, Lenacapavir is distinguished by its multistage mechanism of action and long-acting formulation, requiring only two doses per year. It targets viral assembly and release [25]. However, despite its promising efficacy, resistance mutations to Lenacapavir have been identified, including L56I, M66I, Q67H, K70N, N74D/S and T107N [26,28]. These findings emphasize the necessity for ongoing monitoring of HIVDR in response to this novel drug.

Beyond Lenacapavir, several other non-pol-targeting drug classes have either been approved or are currently under clinical evaluation. Notable among the approved regimens are the CD4 attachment inhibitors Ibalizumab and Fostemsavir. Ibalizumab, a humanized IgG4 monoclonal antibody, was approved by the U.S. Food and Drug Administration (FDA) in 2018. It is administered intravenously and works by blocking HIV entry into CD4 cells [29]. Fostemsavir, approved by the FDA in 2020, is an oral, first-in-class drug that binds HIV-1 gp120, inhibiting its attachment to CD4 cells, and is particularly effective in cases of multidrug resistance [30].

Additionally, Bictegravir, a second-generation HIV IN inhibitor, was approved by the FDA as part of combination therapy. Bictegravir has been reported to have a genetic barrier to resistance similar to DTG and exhibits lower pharmacokinetic risks compared with other IN inhibitors (INSTIs) [31,32]. These attributes highlight its potential for increased future use, potentially surpassing other INSTIs in terms of prescription volume.

Another first-in-class addition to the HIV drug repertoire is the class of maturation inhibitors, which target the HIV Gag protein. Notably, Bevirimat is a key compound in this class that, despite promising results in early clinical trials, was never approved. Its development was discontinued after phase III trials failed to meet primary endpoints, and the emergence of resistance in certain HIV strains, particularly those with mutations in the Gag protein, limited its efficacy [33,36]. Nevertheless, this has provided a window to inform further research into maturation inhibitors.

Novel HIV pol-targeting drugs have recently been proposed, and one notable example is Islatravir, a next-generation NRTI currently undergoing clinical evaluation. As a first-in-class NRTI, Islatravir introduces unique pharmacokinetic properties and a novel mechanism of action, offering the potential for enhanced efficacy against HIV strains resistant to older NRTIs [37,40]. A key feature of Islatravir is its prolonged intracellular half-life, which could facilitate less frequent dosing regimens and [41], in turn, improve patient adherence.

The emergence of novel therapies targeting HIVDR highlights the urgent need for innovative approaches in resistance detection. Among these new treatments, the long-acting formulations stand out for their potential to enhance patient adherence by significantly reducing the pill burden. Concurrently, advancements in resistance detection technologies, such as long-read single-molecule sequencing, offer considerable promise. These techniques not only improve detection accuracy and efficiency but also hold the potential to reduce costs, facilitating broader implementation in clinical settings.

## Sequencing as the preferred clinical diagnostic method for resistance surveillance

Sanger sequencing remains the gold standard method for the detection of HIVDR and is the most used methodology. It is based on di-deoxy chain termination chemistry to generate a representative consensus sequence that constitutes the majority of the viral population [42].

**Fig. 2.**
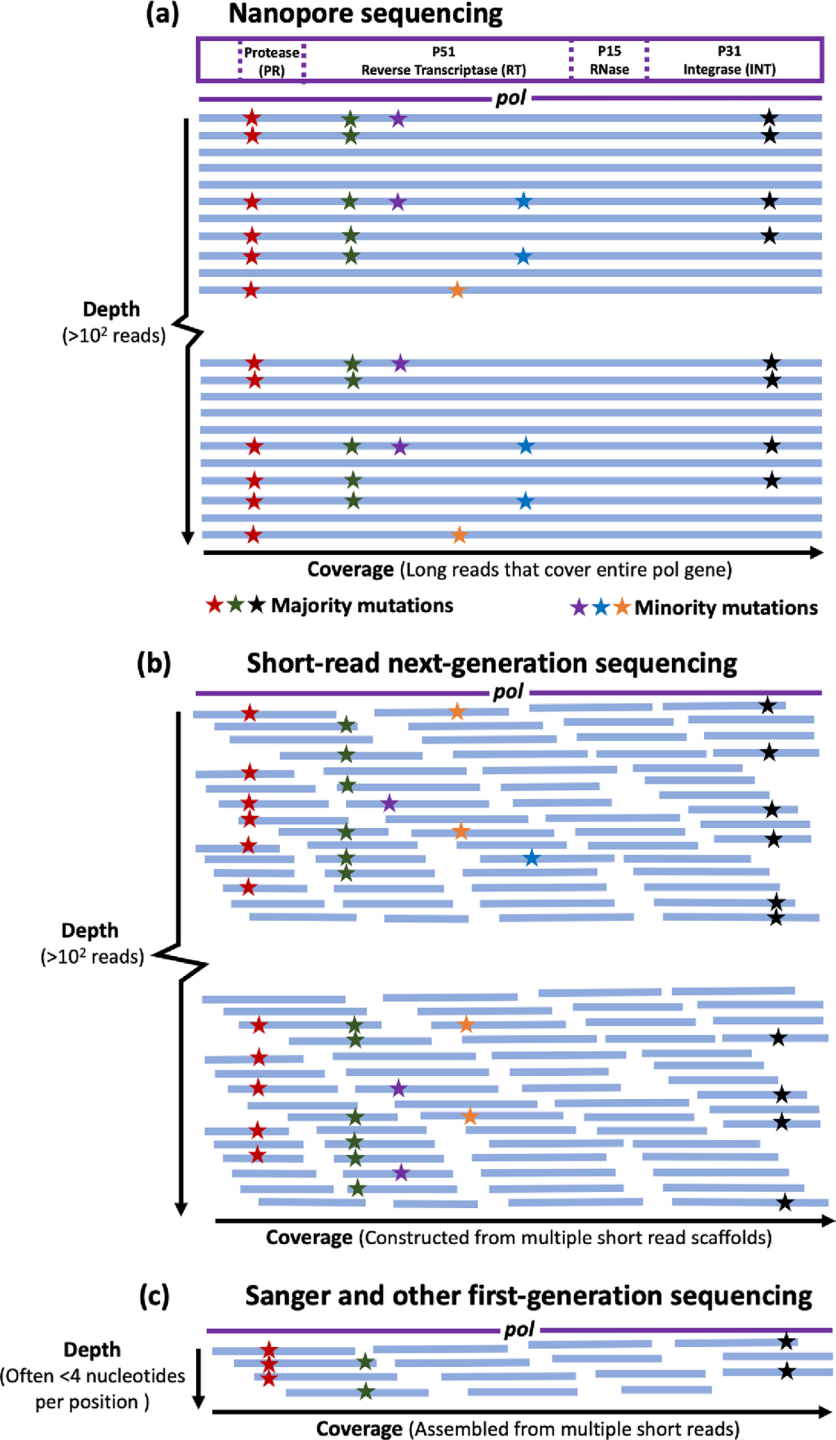
Illustration of nanopore sequencing in comparison to NGS and Sanger sequencing for the detection of HIV-1 drug resistance. The HIV-1 *pol* gene (2843 bp, HXB2 positions 2253–5096) is shown with each horizontal blue line representing a sequencing read, and an asterisk indicates a drug resistance mutation. (**a**) Long-read sequencing such as nanopore sequencing generates continuous reads across the entire *pol* region, minimizing the number of assays and enabling the detection of low-frequency drug resistance. Each read derives from a single molecule, allowing for the identification of linked mutations, such as those represented as red asterisks, which appear in all reads with mutations. This linkage ensures that targeted drugs are effective against all mutant variants, reducing treatment complexity. (**b**) Short-read NGS (e.g. Illumina) lacks mutation linkage information and requires more analytical steps than long-read NGS for equivalent data. (**c**) Sanger sequencing has limited depth, detecting mutations only above a 20% threshold, thereby missing minority variants.

Minority variants present at frequencies below 15–20% are not reliably detected by Sanger sequencing, primarily due to the low coverage (~4 reads per site), limiting its sensitivity for identifying rare variants. Additionally, because Sanger sequencing employs pooled amplification that does not independently process individual molecules, each sequencing read may represent a mixture of populations, contributing to the difficulty of distinguishing minority variants and highlighting two key limitations of this method (Fig. 2). This is of concern since minority resistance variants could be important in the development of HIVDR [43,46]. While Sanger sequencing platforms can sequence fragments up to 900 bp, they suffer from limited data throughput as only 96 reactions can be processed at a time. However, its extensive use for decades has ensured high-quality sequencing data for diagnosing HIVDR in clinical settings. One of the strengths of Sanger sequencing compared with any next-generation sequencing (NGS) platform is the ease with which data can be interpreted and how simple workflows can be implemented to produce highly reproducible data. Despite there being only one FDA-approved assay (ViroSeq, Abbot, Abbot Park, IL, USA) [47,49], which remains expensive, there are a number of commercially available Sanger sequencing assays for HIVDR testing that have been validated and used in resource-limited settings [50]. Although Sanger sequencing is widely validated and has a broad subtype application, it remains a complex and labour-intensive process and is associated with a high capital cost [51].

The advent of NGS methods allows for millions of reads from a single sequencing run to be analysed for HIVDR testing [52]. Although an up-front capital cost is still required to purchase and service large sequencing devices such as Ion-Torrent PGM and Illumina MiSeq, multiplexing sequencing is achievable to reduce costs [53]. Multiple studies have now demonstrated that NGS is a viable alternative for genotypic HIVDR testing, especially for sensitive detection of minority resistance variants, which could lead to treatment failure [53,59]. Yet, NGS methods have only gradually been adopted for HIVDR surveillance, partly due to the computational challenges associated with complex data analysis [60]. Only one commercially available NGS assay has been approved by the FDA for clinical HIVDR testing [61,62]. The Ion-Torrent-based deep sequencing assay has demonstrated high sensitivity and specificity for detecting over 300 HIVDR mutations, although the high cost per sample limits its widespread accessibility for HIVDR surveillance.

Oxford Nanopore Technologies (ONT) produce a range of portable, scalable and affordable sequencing platforms, including the portable USB-powered MinION device [63,64]. Nanopore sequencing passes nucleic acids through a small pore embedded in a membrane. As the nucleic acids pass through the nanopore, they change the electric current, which can be measured and translated into a nucleotide sequence in real time [65,66].

From its commercial launch, the MinION was well poised to be deployed to outbreaks of emerging infectious diseases where there is often a lack of laboratory capacity and sequencing infrastructure, making real-time genomic surveillance challenging [67,70]. Similarly, the release of the Flongle adapter from ONT provides a sequencing platform for smaller, frequent, rapid tests with reduced costs [71]. As a result of its versatility, it has been used in real time during the 2014–2016 Ebola virus disease outbreak in West Africa [70] and during Zika and Yellow fever outbreaks in Brazil [69,72] and enabled a real-time characterization of a Lassa fever outbreak in Nigeria in 2018 [73]. More recently, nanopore sequencing has become embedded into routine clinical diagnostic practice, which improved the management of lower respiratory tract infections [74].

## Nanopore sequencing: a new frontier for expanding HIVDR surveillance

Most of the HIVDR monitoring in LMICs is done in centralized WHO-accredited and monitored facilities [75], often with advanced equipment that requires specialized structured maintenance, and usually their testing turnaround time is in weeks [76]. With low capital cost and versatility, ONT devices such as MinION and Flongle [71,77] make nanopore sequencing well suited for decentralizing HIVDR surveillance in low-resource settings and for training and capacity strengthening. Nanopore sequencing is also portable due to the compact size of the analysers, and it is easy to use due to its adaptability to various pathogens and field environments with minimal alterations across procedures. It also has real-time capability inherent in its design, which permits data analysis at any stage of the sequencing cycle [78]. The real-time sequencing aspect offers the potential for point-of-care testing in clinical settings. The optimal genotypic HIVDR assay must be sensitive, scalable and affordable. Ideally, it should be suitable for all HIV-1 subtypes, regardless of the sample source (e.g. plasma, dried blood spots and cerebral spinal fluid) and capable of sequencing samples with low viral loads. This capability is particularly relevant given the scale-up of ART, especially in LMICs [79,83], the test-and-treat policy for HIV-1 care and the redefinition of clinical failure from ≥1000 to ≥200 copies ml^–1^ [84,87]. These factors have markedly lowered the viral loads of individuals undergoing ART [88,93]. Since viral load is positively associated with the success rates of HIVDR sequencing, lower viral loads reduce these success rates [94,95]. Nanopore sequencing has demonstrated the ability to overcome challenges associated with low PVL, as evidenced by studies on Zika virus, *Escherichia coli* and *Saccharomyces cerevisiae* DNA. These studies highlight the high sensitivity of nanopore sequencing, which can detect as few as 50 genome copies per reaction [69,96], an essential feature for clinical applications such as HIVDR detection. Another benefit of implementing nanopore-based surveillance is its ability to produce long reads derived from a single molecule, which can allow for the identification of HIVDR mutations on the same virion [97] and will allow for integrated detection of resistance profiles, reducing the number of genotyping assays, hence improving diagnostics and patient management. This is particularly important considering the development of new long-acting HIV drugs, including novel classes whose HIVDR profiles are not well documented. Such drugs include, though not exclusively, Lenacapavir, a first-in-class HIV capsid inhibitor [20,22], Fostemsavir, also a first-in-class CD4-attachment inhibitor [30], Bictegravir, a second-in-class IN inhibitor [31,32], Beviramat, a first-in-class maturation inhibitor [33,36], and Islatravir, a next-generation long-acting NNRTI [37,39, 40]. The number of HIVDR mutations monitored for clinical management has grown significantly in recent years [98] (Table 1). Introducing new drug classes underscores the increasing need for advanced sequencing technologies, such as nanopore sequencing, to comprehensively monitor these mutations.

**Table 1.** HIVDR mutations monitored for clinical management [98] This table outlines the HIVDR mutations that are commonly monitored for clinical management, detailing their respective drug classes and the impact of these mutations on the susceptibility of various drug regimens. The Gene column indicates the associated HIV gene: PR, RT or IN. The Mutation column lists the wild-type amino acid, its position within the gene and the corresponding mutant amino acid(s). The Drug class column specifies the relevant classes of antiretroviral drugs: NRTIs, NNRTIs and INSTIs.

Protein	Mutation name	Associated drug class	Affected drug susceptibility
		**Protease**	
PR	L10F	Protease inhibitor (PI)	Atazanavir
	K20T		
	L24I		
	V32I		
	L33F		
	M46I/L		
	G48V		
	I50L		
	F53L/Y		
	I54L/V/M/T/A/S		
	G73C/S/T/A		
	V82A/T/F/L/M/S		
	I84V		
	I85V		
	N88S		
	L90M		
PR	V11I	Protease inhibitor (PI)	Darunavir/Ritonavir
	V32I		
	L33F		
	I47V		
	I50V		
	I54M/L		
	T74P		
	L76V		
	I84V		
	L89V		
PR	L10F/I/R/V	Protease inhibitor (PI)	Lopinavir/Ritonavir
	K20M/R		
	L24I		
	V32I		
	L33F		
	M46I/L		
	I47V/A		
	150V		
	F53L		
	I54V/L/A/M/T/S		
	A71V/T		
	G73S		
	L76V		
	V82A/F/T/S		
	I84V		
	I90M		
PR	L10V	Protease inhibitor (PI)	Tipranavir/Ritonavir
	L33F		
	M36I/L/V		
	K43T		
	M46L		
	I47V		
	I54A/M/V		
	Q58E		
	H69K/R		
	T74P		
	V82L/T		
	N83D		
	I84V		
	L89I/M/V		
PR	L10F/I/R/V	Protease inhibitor (PI)	Fosamprenavir/Ritonavir
	V32I		
	M46I/L		
	I47V		
	I50V		
	I54L/V/M		
	G73S		
	L76V		
	V82A/F/S/T		
	I84V		
	L90M		
PR	L10I/R/V	Protease inhibitor (PI)	Indinavir/Ritonavir
	K20M/R		
	L24I		
	V32I		
	M36I/L/V		
	M46I/L		
	I54/V		
	A71V/T		
	G73S/A		
	I76V		
	V77I		
	V82A/F/T		
	I84V		
	I90M		
PR	L10F/I	Protease inhibitor (PI)	Nelfinavir
	D30N		
	M36I		
	M46I/L		
	A71V/T		
	V77I		
	V82A/F/T/S		
	I84/V		
	N88D/S		
	L90M		
	L10I/R/V	Protease inhibitor (PI)	Saquinavir/Ritonavir
	L24I		
	G48V		
	I54V/L		
	I62V		
	A71V/T		
	G73S		
	V77I		
	V82A/F/T/S		
	I84V		
	L90M		
		**Reverse Transcriptase**	
RT	M41L	NRTI	Multi-NRTI Resistnce
	A62V		
	▼69Insert		
	k70R		
	L210W		
	T215Y/F		
	K219Q/E		
RT	A62V	NRTI	Multi-NRTI Resistnce except Tenofovir
	V75I		
	F77L		
	F116Y		
	Q151M		
RT	M41L	NRTI	Multi-NRTI Resistnce except Emtricitabine and Lamivudine
	K70R		
	L210W		
	T215Y/F		
	K219Q/E		
RT	K65R/E/N	NRTI	Abacavir
	L74V		
	Y115F		
	M184V		
RT	K65R/E/N	NRTI	Emtricitabine/Lamivudine
	M184V		
RT	K65R/E/N	NRTI	Tenofovir
	K70E		
RT	M41L		Zidovudine
	D67N		
	K70R		
	L210W		
	T215Y/F		
	K219Q/E		
RT	K65R/E/N	NRTI	Didanosine
	L74V		
RT	M41L	NRTI	Stavudine
	K65R/E/N		
	D67N		
	k70R		
	L210W		
	T215Y/F		
	K219Q/E		
RT	V106A/I/M/T	NNRTI	Doravirine
	Y188L		
	G190E		
	P225H		
	P227/C/I/L/R/V		
	M230L		
	L234I		
	Y318F		
RT	L100I	NNRTI	Efavirenz
	K101P		
	K103N/S		
	V106M		
	V108I		
	Y181C/I		
	Y188L		
	G190S/A		
	P225H		
	M230L		
RT	V90I	NNRTI	Etravirine
	A98G		
	L100I		
	K101E/H/P		
	V106I		
	E138A/G/K/Q		
	V179D/F/T		
	Y181C/I/V		
	G190S/A		
	M230L		
RT	L100I	NNRTI	Neverapine
	K101P		
	K103N/S		
	V106A/M		
	V108I		
	Y181C/I		
	Y188C/L/H		
	G190A		
	M230L		
RT	L100I	NNRTI	Rilpivirine
	K10IE/P		
	E138A/G/K/Q/R		
	V179L		
	Y181C/I/V		
	Y188L		
	H221Y		
	F227C		
	M230I/L		
		**Integrase**	
IN	G118R	INSTI	Bictegravir
	E138A/K/T		
	G140A/C/R/S		
	Q148H/K/R		
	S153F/Y		
	R263K		
IN	T66K	INSTI	Cabotegravir
	T97A		
	G118R		
	E138A/K/T		
	G140A/C/R/S		
	Q148H/K/R		
	S153F/Y		
	N155H		
	R263K		
IN	G118R	INSTI	Dolutegravir
	E138A/K/T		
	G140A/C/R/S		
	Q148H/K/R		
	S153F/Y		
	N155H		
	R263K		
IN	T66I/A/K	INSTI	Elvitegravir
	E92Q/G		
	T97A		
	F121Y		
	S147G		
	Q148H/K/R		
	N155H		
	R263K		
IN	L74M	INSTI	Raltegravir
	E92Q		
	T97A		
	F121Y		
	E138A/K		
	G140A/S		
	Y143R/H/C		
	Q148H/K/R		
	N155H		
	R263K		
		**Gag**	
GAG	L56I	Capsid Inhibitors	Lenacapavir
	M661		
	Q67H		
	K70N/S/R		
	N74/D/S		
	A105T		
	T107N		
		**Envelope**	
ENV	G36D/S	Entry Inhibitor	Enfuvirtide
	I37V		
	V38A/M/E		
	Q39R		
	Q40H		
	N42T		
	N43D		

One of the major concerns with adopting ONT for resistance testing is systematic errors in reads, which are mainly attributed to indel errors in homopolymer regions, where multiples of the same nucleotide appear consecutively. This is particularly important for common drug resistance-associated mutations, such as K65R and K103N, which occur in homopolymer tracts [56,99, 100]. A read error in these regions could lead to the false detection of these mutations, even though they are absent. However, these errors can be reduced by error correction tools and machine-learning algorithms for post-assembly polishing, such as Homopolish, NextPolish, CANU, Apollo, Raven, HGAP and Medaka [101,107]. A recent analysis of bacterial genomes found that the vast majority of homopolymers are correctly resolved up to a length of 11 bp in R10.4 data [108] with median read accuracy at ~99.1% (Q20) [109]. Despite the availability of multiple sequence polishing algorithms, the high per-base error rate of raw data remains a significant challenge for nanopore sequencing in HIV long-read analysis, primarily due to the limited data on the application of these polishing algorithms to HIV genomes. To address this, a novel pipeline, HMMPolish, was introduced to enhance genome accuracy by focusing on correcting protein-coding regions in RNA virus genomes derived from long-read sequencing. HIV and other RNA viruses, which lack stringent proofreading mechanisms, are prone to replication-induced mutations that complicate viral sequence analysis [110]. HMMPolish was tested on a real ONT dataset from HeLa cells infected with HIV-1 and compared with other polishing tools. The results showed that HMMPolish outperformed all other tools in correcting errors in protein-coding regions, with fewer gaps and mismatch errors, particularly in the Gag and Pol proteins. HMMPolish’s reliance on viral protein families made it highly effective for polishing known RNA virus genomes, though it is not suitable for newly discovered viruses without established protein HMMs.

## Advancements in nanopore sequencing for HIVDR detection

HIVDR detection workflows traditionally focus on the PR and RT genes, but the growing use of IN inhibitors has made the IN gene a frequent target. Nanopore sequencing is well suited for sequencing larger portions of the HIV genome. Studies using recent nanopore flowcell versions (R10.4.1) and V14 chemistry have achieved up to 99.9% accuracy, with clinical validation showing 92.5% concordance for HIVDR genotypes and 98.7% for tropism compared with Sanger sequencing [59]. Partial sequencing of the *pol* gene at low sequence coverage, analysed with the Nano-RECall workflow, achieved a 99.3 and 99.6% sequence similarity with Sanger sequences for subtype C viruses [56]. Further improvements include CODEHOP-mediated PCR primers, which reduce bias from consensus and degenerate primers and improve PCR success rates to 97–98% compared with 82–84% with standard primers [111]. Nanopore sequencing has been effective in resource-limited settings, including a study in Angola, which found no major IN mutations in over 40 samples, though accessory IN mutations were detected, along with major NRTI, NNRTI and PI mutations [112]. In North America, portable nanopore workflows showed high concordance with PacBio sequencing in detecting HIVDR, particularly in low viral load samples below 1000 copies ml^–1^ [55], supporting the feasibility of sequencing low viral load samples, which is now a common occurrence due to the expanded ART rollout. Nanopore sequencing also has utility in epidemiological surveillance, where the full-length HIV-1 genome structure of recombinant forms has been identified [113].

The increasing adoption of NGS for HIVDR detection has sparked discussions about the need for quality assurance programmes to standardize sequencing procedures in clinical diagnostic laboratories. The Second Winnipeg Consensus Symposium addressed global readiness for NGS-based HIVDR detection, examining progress and challenges for broader implementation [60]. A variety of sequencing protocols and bioinformatics analyses have been developed across different groups [114,115], highlighting the need for standardized consensus guidelines for routine clinical care. The inaugural Winnipeg Consensus meeting in 2018 focused on bioinformatics requirements and proposed a consensus for NGS data analysis in HIVDR, aiming to address variations arising from the analysis of mutations below the 1% threshold, which are not a concern at higher mutation abundances [114]. The symposium underscored the necessity for continued research to develop robust recommendations for NGS-based HIVDR detection.

## Implementation of nanopore sequencing for resistance profiling in other infectious diseases

Real-time genomics, driven by nanopore sequencing, has the potential to significantly speed up antibiotic resistance profiling directly in clinical settings [116]. For example, it has enabled the rapid identification of drug-resistant genes in *Mycobacterium tuberculosis* (MTB) genomes, reducing turnaround times to mere hours, which is in stark contrast to traditional tests, which can require days or even weeks for culturing [117]. The use of a targeted nanopore sequencing assay (NanoTB^®^) was recently endorsed by the WHO for the detection of drug-resistant tuberculosis. This marks a paradigm shift in the diagnosis of drug-resistant MTB. One study evaluating this targeted MTB nanopore sequencing assay demonstrated its good performance, flexibility and reduced testing times compared with other existing solutions [118]. Additionally, other case studies on MTB have highlighted nanopore sequencing’s ability to deliver highly accurate SNP calls and reliably predict drug resistance [119,120]. This technology has also proven to be viable for genomic surveillance of *Plasmodium falciparum*, the parasite responsible for malaria, particularly in endemic regions [121]. By employing a multiplexed PCR approach to target key antimalarial resistance markers, Girgis *et al*. [122] have produced rapid, accurate and cost-effective data using a custom Nextflow pipeline. Collectively, these studies emphasize the growing shift towards nanopore sequencing in the field of infectious disease, especially for antimicrobial resistance profiling.

## Bioinformatic considerations for resistance profiling

Historically, the analysis of HIVDR has primarily depended on the Stanford University HIV Drug Resistance Database (HIVdb) algorithm, which provides a user-friendly interpretation of HIV resistance data [123]. Several web-based data analysis pipelines are freely available for analysing Sanger and Illumina sequencing data, including RECall, HyDRA, PASeq and MiCall [50,54, 99, 124]. While these tools have proven to be effective in generating consistent, easily interpretable and rapid results, they raise concerns about patient privacy due to the transmission of sensitive data across international networks. Moreover, they generally do not support the analysis of nanopore sequencing data. Conventional nanopore sequencing analysis is comparable in technical complexity to Sanger sequencing but offers distinct advantages in automation, for HIVDR detection, due to the availability of full-quality scores, and higher depth per site that gives more support for variant detection. However, it demands advanced computational expertise, often exceeding the capabilities of most users, thus requiring specialized training or bioinformatics proficiency for effective implementation. To address this, ClusterV-Web, a user-friendly web application, was developed to simplify the analysis process by providing an accessible platform specifically designed for long-read HIVDR analysis [57]. However, the high throughput of nanopore data, combined with the error rate and the genetic diversity of HIV, makes downstream analysis challenging. While high read coverage for conserved regions can mitigate most sequencing errors with the help of reference genomes and computational tools, systematic issues, particularly homopolymer-induced errors, still pose challenges [115,125,128]. To address residual errors, the NanoHIV pipeline was introduced, employing an iterative consensus approach for analysing near-full-length HIV nanopore data [53]. This method, validated with single-genome sequences and benchmarked against Illumina NGS data, achieved an average agreement of 99.4% [53]. The Nano-RECall pipeline has also been designed to correct for ONT homopolymer-associated read errors but is currently limited to highly abundant drug resistance variants from subtype C viruses [56].

Containerization software such as Shifter [129], Docker [130] and Singularity [131], along with workflow managers like Nextflow [132] and Snakemake [133], offer excellent options for developing streamlined and user-friendly nanopore analysis pipelines. For example, docker containers such as HIVseqDB [134] and Quasiflow [135] already exist for the secure analysis of NGS-based HIVDR data. Furthermore, pipelines can be enhanced by integrating the Stanford HIVdb interpretation system locally via the Sierra web service. An open-source implementation of the Stanford HIVdb genotypic resistance interpretation system has been developed, which allows for local execution circumventing ethical, legal and infrastructure concerns that arise from relying on remote computing [136]. Regardless of the technical solution, future workflow technologies should align with the FAIR4RS principles to foster a more robust and sustainable workflow community [137].

Read length can be leveraged to enhance the analysis metrics of sequencing data, as demonstrated in shotgun metagenomics studies utilizing short-read, long-read and hybrid sequencing approaches with Illumina and PacBio platforms, each offering distinct advantages. Long-read sequencing excels in assembly quality, short-read sequencing is superior for bin refinement and hybrid approaches provide the longest assemblies and highest mapping rates, with the optimal strategy being context-dependent [47]. Furthermore, read length analysis strategies, such as using short reads to correct long-read accuracy, as demonstrated with PacBio [138,139], may also be applied to nanopore reads to improve mapping quality and alignment sensitivity [115].

## Future directions

Nanopore sequencing holds great promise as a powerful tool for assessing HIVDR, and its versatility is further proven by its successful application across a wide range of infectious diseases. The COVID-19 pandemic has driven a surge in genome-sequencing capacity, particularly across regions in Asia and Africa, supported by decentralized sequencing workflows for genomic surveillance. There are opportunities to leverage this investment and capacity while also capitalizing on the technological upgrades that ONT has made in its chemistry, flowcells and basecalling models. However, several challenges remain for the widespread application of nanopore sequencing for HIVDR genotyping, necessitating further research and development. It is also worthwhile to note that nanopore sequencing remains an evolving technology, with recurrent updates to kits, reagents, basecalling models and software tools, rendering older versions obsolete. This poses considerable challenges for diagnostic and surveillance laboratories, as validated methods can rapidly become outdated, requiring re-validation with new reagents, an issue that warrants attention. Nevertheless, as laboratory protocols for HIVDR continue to evolve, there is an urgent need for sophisticated, open-source bioinformatics workflows to manage the vast amount of sequence data, ensuring its reliability and clinical relevance.

## Abbreviations

ART: antiretroviral treatment
DTG: Dolutegravir
FDA: U.S. Food and Drug Administration
IN: integrase
INSTI: integrase strand transfer inhibitor
LMIC: low- and middle-income country
MTB: *Mycobacterium tuberculosis*
NGS: next-generation sequencing
NNRTI: non-nucleoside reverse transcriptase inhibitor
NRTI: nucleoside reverse transcriptase inhibitor
ONT: Oxford Nanopore Technologies
PDR: pre-treatment HIV drug resistance
PI: protease inhibitor
PR: protease
PVL: plasma viral load
RT: reverse transcriptase
WHO: World Health Organization
